# Case Report: A Pediatric Case of Lipoprotein Glomerulopathy in China and Literature Review

**DOI:** 10.3389/fped.2021.684814

**Published:** 2021-08-27

**Authors:** Yue Song, Changqiang Yang, Lan Liu, Hua Wang

**Affiliations:** ^1^Department of Pediatrics, West China Second University Hospital, Sichuan University, Chengdu, China; ^2^Key Laboratory of Birth Defects and Related Diseases of Women and Children, Ministry of Education, Sichuan University, Chengdu, China; ^3^Department of Cardiology, West China Hospital, Sichuan University, Chengdu, China

**Keywords:** lipoprotein glomerulopathy, pediatric, Chinese, nephrotic syndrome, anemia

## Abstract

**Background:** Lipoprotein glomerulopathy is a rare kidney disease characterized by lipoprotein thrombi in the glomerulus. Here, we report a case of lipoprotein glomerulopathy in a Chinese pediatric patient. Furthermore, we summarized the clinical features and genetic characteristics of lipoprotein glomerulopathy in China.

**Case Presentation:** An 11-year-old Chinese girl presented with nephrotic syndrome with anemia (98 g/L). After excluding secondary causes, primary nephrotic syndrome was considered. Treatment with prednisone (60 mg/day) did not improve her condition. Renal biopsy showed marked dilation of the capillary lumen with lipoprotein thrombi and positive oil red O staining. Genetic testing revealed the genetic variant c.127C > T (p.R43C), known as the Kyoto mutation of the APOE gene. These findings are consistent with the diagnosis of lipoprotein glomerulopathy. Prednisone was gradually tapered and captopril was initiated. A 2-year follow-up revealed elevated urine protein and serum creatinine levels. We also reviewed 17 pediatric and 156 adult cases of lipoprotein glomerulopathy reported in China from the year of creation to 2021. The most common clinical features were edema, hematuria, hypertriglyceridemia, and increased serum apoE levels. Extra-renal manifestations included anemia, splenomegaly, and cardiac lipoprotein deposition.

**Conclusion:** APOE Kyoto is the most common mutation in patients with lipoprotein glomerulopathy. In China, homozygosity for E3 (E3/3) is the most common isoform.

## Introduction

Lipoprotein glomerulopathy (LPG) is a rare hereditary renal disorder associated with abnormal lipid metabolism induced by variations in plasma apolipoprotein E (apoE) (1, 2). Human apoE is a 34-kDa protein with 299 amino acids that mediate the uptake of triglyceride-rich lipoproteins through both the low-density lipoprotein cholesterol (LDL) receptor and LDL receptor-related protein pathways (3). LPG always presents with proteinuria or nephrotic syndrome (NS). The most important histopathologic manifestation of LPG is the presence of massive eosinophilic lipoprotein thrombi within dilated glomerular capillary lumen with positive oil red O staining (1). The prognosis of patients with LPG is poor due to the absence of specific treatment. Approximately 50% of patients progress to end-stage renal disease within 1–27 years of onset (4). In patients who underwent kidney transplantation, relapse was observed from 5 months to 2 years of follow-up (5).

In China, pediatric LPG cases have rarely been reported. Here, we report a case of LPG in an 11-year-old female with no known family history of renal disease who was admitted to our hospital for a 1-month history of mild edema of the lower limbs. Furthermore, we summarized the clinical features and genetic characteristics of LPG cases in China.

## Case Description

An 11-year-old Chinese girl was admitted to our hospital who presented with nephrotic syndrome. She weighed 49 kg and her blood pressure was 120/80 mmHg. Physical examination at admission revealed mild edema of the lower limbs with no associated corneal arcus or xanthoma. Laboratory investigations showed anemia (98 g/L), elevated serum urea (6.7 mmol/L) and creatinine (58 μmol/L), and decreased albumin (21 g/L). Urinalysis revealed proteinuria (4+) and microscopic hematuria (3+). Urinary protein was 2.06 g/24 h. Lipid profile showed elevated total cholesterol (5.04 mmol/L), elevated triglyceride (2.18 mmol/L), and normal LDL-cholesterol (2.24 mmol/L).

Based on laboratory findings, secondary causes of NS were excluded. Anti-nuclear, anti-cardiolipin, anti-neutrophil cytoplasmic, and anti-glomerular basement membrane antibodies were negative. Rheumatoid factor was also negative. Hepatitis B and C serology were negative. In addition, complement C3 and C4 levels were normal. Renal ultrasonography revealed normal-sized kidneys. These findings were consistent with the diagnosis of primary NS.

**Figure 1:**
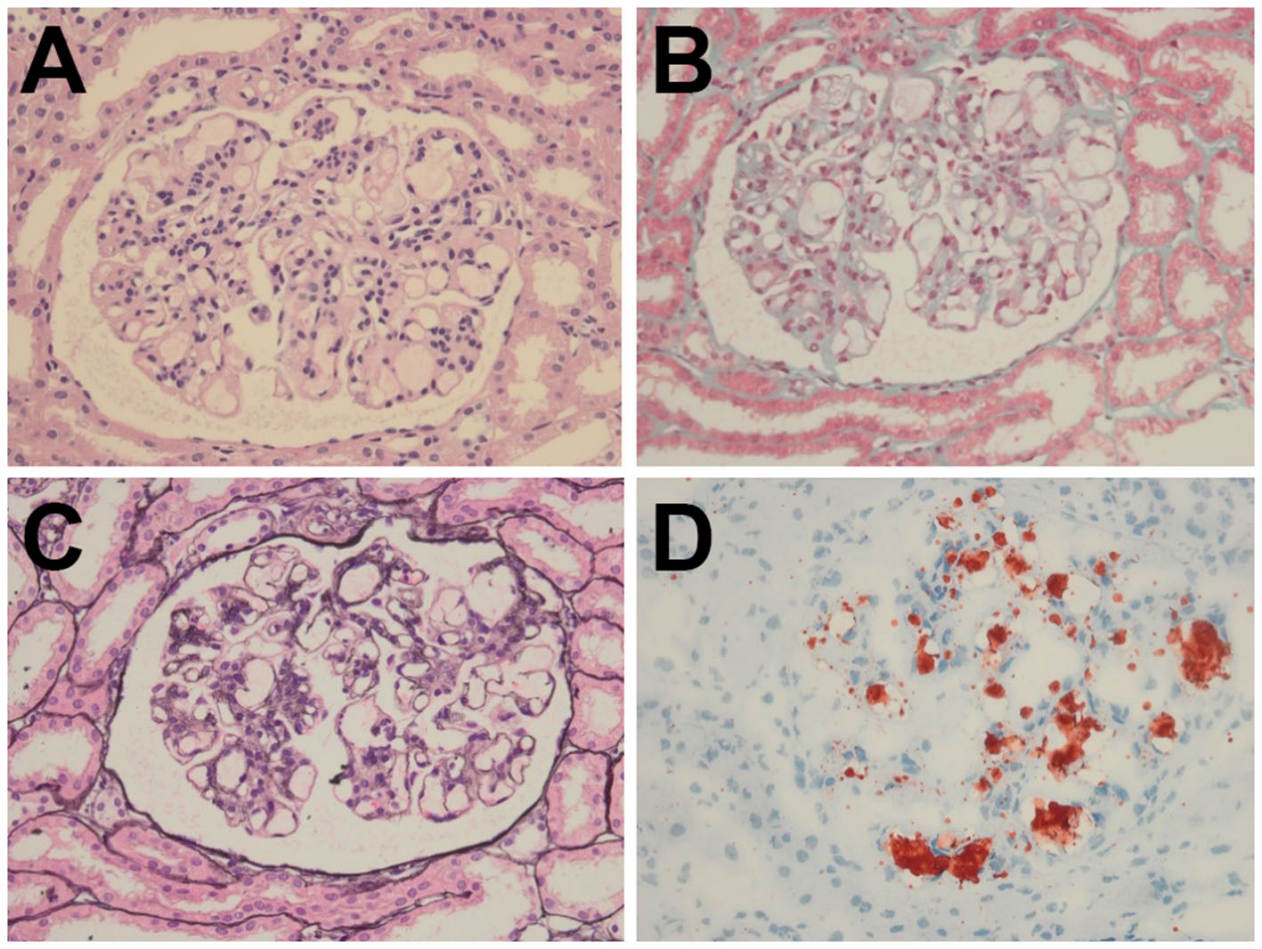
Histopathology of the renal biopsy specimen. **(A)** H&E (× 400) and **(B)** Masson (× 400) staining showed segmental glomerulosclerosis with mild-to-moderate proliferation of the mesangial cells and matrix. **(C)** PASM (× 400) staining revealed marked dilatation of the capillary lumina with lipoprotein thrombi. **(D)** Oil Red O (× 400) staining confirmed the presence of intraluminal lipid droplets.

Despite treatment with prednisone (60 mg/day) and captopril (60 mg/day) for 2 months, her massive proteinuria and microscopic hematuria persisted, thereby prompting further evaluation *via* percutaneous renal biopsy. Light microscopy showed segmental glomerulosclerosis in three out of nine samples and mild-to-moderate proliferation of the mesangial cells and matrix (Figures 1A,B). The capillary lumina were dilated due to the presence of lipoprotein thrombi with pale staining on periodic acid-methenamine silver (Figure 1C). Oil red O staining confirmed the presence of intraluminal lipid droplets (Figure 1D). Immunofluorescence microscopy revealed that immunoglobulins (i.e., IgG, IgA, and IgM) and complement factor 1q (C1q) were negative. These findings were highly suggestive of LPG.

**Figure 2:**
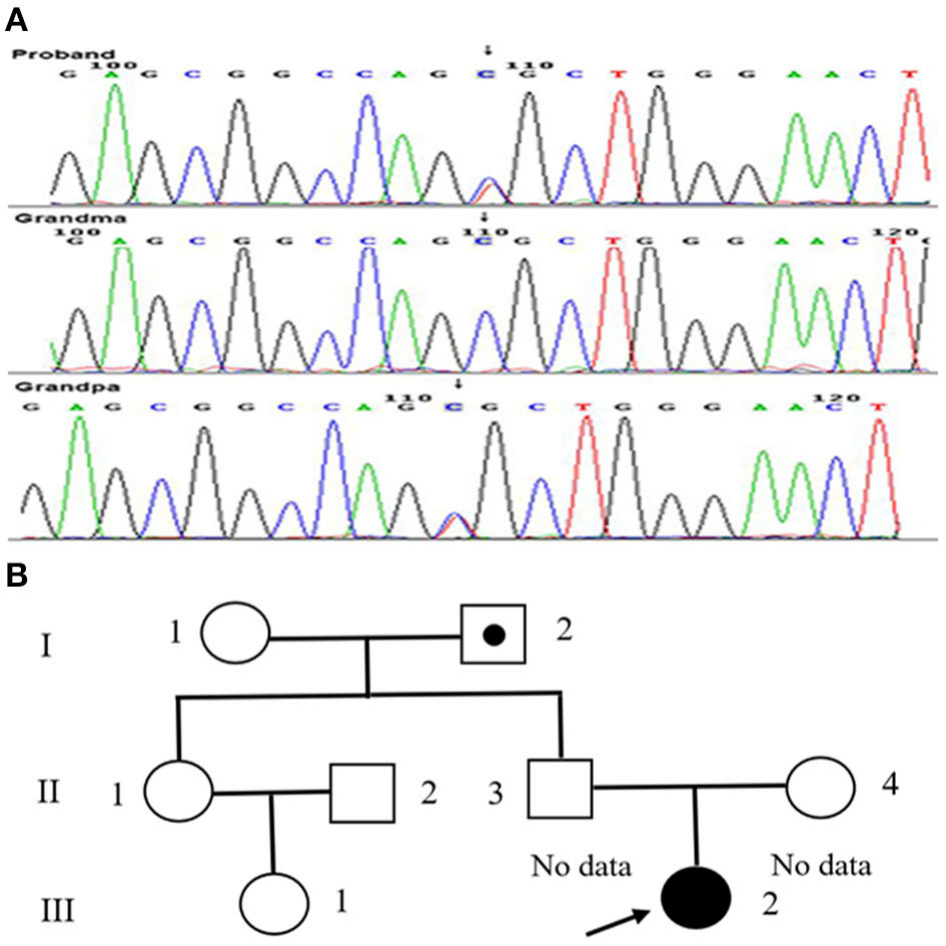
Mutation dictation of APOE and family pedigree of our patient. **(A)** The mutation in the patient (arrow). **(B)** The family pedigree of the patient. Proband III-2 is the present patient (arrow). Circles and squares indicate the females and males, respectively. The dotted symbol represents asymptomatic LPG carriers. II-1 and II-2 did not undergo genetic testing for special reasons, both of whom had no history of renal disease.

Genetic testing confirmed the presence of the c.127C>T (p.R43C) or Kyoto mutation of the APOE gene (Figure 2A). Her parents refused to undergo genetic screening. Therefore, her grandparents were investigated and underwent genetic screening. Her grandfather was asymptomatic and her grandmother was otherwise healthy. The APOE mutation in the patient and her grandfather were heterozygous (Figure 2B). In addition, the patient did not have any siblings.

All of these findings were consistent with the diagnosis of LPG. Prednisone was gradually tapered. Ideally, the patient should have been given captopril and a lipid-lowering agent. However, her grandfather refused the latter.

A 2-year follow-up revealed that her serum creatinine (102 μmol/L) and urine protein levels (3.2 g/24 h) were elevated. Moreover, her lipid profile showed elevated total cholesterol 6.56 mmol/L, triglyceride (2.82 mmol/L), and LDL-cholesterol (3.87 mmol/L).

## Discussion

LPG is a rare autosomal dominant renal disease. Although an increasing number of LPG cases have been reported in China, pediatric LPG cases are rare. We described a case of LPG in an 11-year-old girl initially presenting with nephrotic syndrome and anemia. Based on the laboratory examination, there was no evidence suggestive of a secondary cause of NS. However, initial treatment with prednisone did not improve her NS, prompting further evaluation with renal biopsy and genetic testing.

Furthermore, we conducted a literature search of Chinese LPG cases from various databases (i.e., China National Knowledge Infrastructure, Wanfang, WeiPu, PubMed, and Web of Science) from the year of creation to 2021. The language was limited to Chinese and English. Including our case, a total of 18 pediatric cases and 156 adult cases were identified. The retrieved clinical data of these cases are summarized in Tables 1, 2. There were 18 pediatric patients (male = 13; female = 5) with an average age of 12.0 ± 2.90 years (range, 7–17). In contrast, there were 156 adult patients (male = 87; female = 69) with an average age of 35.6 ± 13.8 years (range, 18–72). Furthermore, LPG was mainly diagnosed in teenagers aged 11–17 years and adults aged 31–50 years.

**Table 1:** Age and sex distribution of 96 patients with LPG in China.

	**Age at onset (years)**						
**Gender**	**≤10**	**11–17**	**18–30**	**31–50**	**51–65**	**>65**	**Total**
Male	2	11	15	24	4	0	56
Female	2	3	8	22	4	1	40
Total	4	14	23	46	8	1	96

**Table 2:** Clinical features and genetic characteristics of Chinese LPG patients.

**Variable**	**Children (** ***n*** **=** **18)**		**Adult (** ***n*** **=** **156)**		
	**Number of patients with available data**	**Number of positive patients**	**Number of patients with available data**	**Number of positive patients**	**Percentage, *n*/*N* (%)**
Edema (*n*)	14	11	71	70	81/85 (95.3)
Hypertension (*n*)	11	8	107	76	84/118 (71.2)
Hematuria (*n*)	10	9	124	109	118/134 (88.1)
Anemia (*n*)	10	9	50	30	39/60 (65.0)
Albumin <30 g/L	11	6	67	36	42/78 (53.8)
Elevated Scr (μmol/L)	13	5	72	8	13/85 (15.3)
TC > 5.7 mmol/L	13	6	87	54	60/100 (60.0)
TG ≥ 1.7 mmol/L	13	10	88	79	89/101 (88.1)
LDL > 3.12 mmol/L	10	6	57	23	29/67 (43.3)
apo B > 1.33 g/L	9	4	44	12	16/53 (30.2)
apo E ≤ 4.5 mg/dl	11	0	47	6	6/58 (10.3)
apo E > 4.5 mg/dl	11	11	47	41	52/58 (89.7)
Normal lipid levels (*n*)	8	0	44	6	6/52 (11.5)
**24 h Upro (g/day)**					
0.5–1.0	15	0	71	5	5/86 (5.81)
1.0–3.5	15	5	71	19	24/86 (27.9)
>3.5	15	10	71	48	58/86 (67.4)
**apo E Isoforms**					
E2/E3	6	0	89	11	11/95 (11.6)
E3/E3	6	2	89	50	52/95 (54.7)
E3/E4	6	3	89	23	26/95 (27.4)
?/E4	6	1	89	0	1/95 (1.1)
E3/?	6	0	89	2	2/95 (2.1)
No isoforms identified	6	0	89	3	3/95 (3.3)
**APOE mutations**					
Kyoto (R25C)	9	4	86	49	53/95 (55.8)
E4 (C112A)	9	0	86	2	2/95 (2.1)
Tokyo (del 141–143)	9	1	86	2	3/95 (3.2)
Maebasi (del 142–144)	9	3	86	5	8/95 (8.4)
APOE (143 K-147R)	9	0	86	2	2/95 (2.1)
Chicago (A147P)	9	0	86	1	1/95 (1.1)
Shenzhen (A150C)	9	0	86	1	1/95 (1.1)
Guangzhou (Al50P)	9	0	86	4	4/95 (4.2)
E2(A158C)	9	0	86	1	1/95 (1.1)
Chengdu (L173P)	9	0	86	1	1/95 (1.1)
Osaka/Kurashiki (R176P)	9	0	86	3	3/95 (3.2)
Hong Kong (A230T)	9	0	86	1	1/95 (1.1)
No mutations identified	9	1	86	15	16/95 (16.8)

*Hb, Hemoglobin; apo B, Apolipoprotein B; apo E, Apolipoprotein E; TG, triglyceride; TC, total cholesterol; HDL-C, high-density lipoprotein cholesterol; LDL, low-density lipoprotein cholesterol; Upro, urinary protein. Scr, serum creatinine. The definitions of hypertension, elevated Scr, and anemia were based on the reference values of different age groups of patients according to guidelines in China*.

The most common clinical features were edema (95.3%), hematuria (88.1%), hypertriglyceridemia (88.1%), and increased serum apoE levels (89.7%). Other common clinical features were hypertension (71.2%) and anemia (65.0%). However, there were six patients with no clinical signs of systemic hyperlipidemia (e.g., xanthomas and corneal arcus). Furthermore, plasma lipid levels were within normal ranges in these patients (6–8). These findings indicate that the disease is localized primarily in the glomerulus due to the presence of lipoprotein thrombi (9). We also observed that 39 patients presented with unexplained anemia, especially 20 patients with Hb <90 g/L. Among them, one patient presented with severe anemia (Hb 38 g/L) likely due to severe thrombotic microangiopathy (10).

In our case, the patient also had mild anemia, suggesting that this might be an extrarenal manifestation of LPG. In addition, unexplained splenomegaly has been reported in patients with LPG (11, 12). Morris et al. (13) also reported that intravascular cardiac lipoproteinosis may be an extrarenal manifestation of LPG. However, the exact pathophysiologic mechanism of these extrarenal manifestations remains unclear.

The *APOE* gene is located on chromosome 19q13.2. It is polymorphic with three alleles (i.e., E2, E3, and E4), creating six genotypes (i.e., E2/2, E3/3, E4/4, E2/3, E2/4, and E3/4) (14). In Chinese patients with LPG, homozygosity for E3 (E3/3) is the most common isoform. LPG-associated apoE3 mutations play central roles in the pathogenesis of LPG through protein misfolding, thermodynamic destabilization, and aggregation (15). In the Chinese population, 12 *APOE* mutation sites have been reported to date, with *APOE* Kyoto mutation being the most common. Mutations in *APOE* Chengdu (p.L173P) (16), *APOE* Guangzhou (p.Rl50P) (17), *APOE* Shenzhen (p.R150C) (18), and *APOE* Hong Kong (p.D230Y) (19) have been reported.

In our case, the patient and her grandfather had the same *APOE* Kyoto mutation. However, her grandfather did not present with the clinical features of LPG. There are two possible explanations for this phenomenon. First, the *APOE* mutation could have been transmitted as autosomal dominant with incomplete penetrance (17, 20, 21). Second, the Kyoto mutation could have reduced the clearance of apoE from the circulation, requiring a “second hit” before LPG develops (22).

DNA analysis of two LPG patients revealed the co-existence of *APOE* Kyoto (p.R25C) with E4 (p.C112R) (23) and Hong Kong (p.D230Y) mutations (12). This may have a cumulative effect and ultimately result in phenotypic expression similar to LPG (12). However, 1 pediatric and 15 adult LPG cases did not present with any mutations. In *APOE* knockout mouse models, Wen et al. (24) demonstrated that *APOE* Sendai mutation was not necessarily required for the development of LPG. Therefore, *APOE* mutation is not a necessary factor in the pathogenesis of LPG. However, the underlying mechanisms of LPG remain unclear.

The various treatment strategies done in Chinese LPG cases are presented in Table 3. For patients with LPG, glucocorticoids and immune suppressants were ineffective. The efficacy of lipid-lowering agents is controversial because the types, dosages, courses, and evaluation were variable among different studies (7, 25–27). In addition, few case reports have shown that double-filtration plasmapheresis therapy may be effective (23).

**Table 3:** Treatment strategy of Chinese LPG patients.

**Variable**	**Age at onset**	
	**≤18 years, *n*/*N* (%)**	**>18 years, *n*/*N* (%)**
Lipid-lowering agents	4/5 (80%)	90/108 (83.3%)
Staphylococcal protein-A immunoadsorption	0/5 (0%)	13/108 (12.0%)
Double filtration plasmapheresis	1/5 (20%)	2/108 (1.9%)
Renal transplantation	0/5 (0%)	3/108 (2.8%)

Moreover, Xin et al. (28) administered staphylococcal protein-A immunoadsorption in 13 patients with LPG for 10 sessions of 10 cycles per session. After treatment, urine protein and serum apoE levels decreased. Moreover, the intraglomerular lipoprotein thrombi almost disappeared. Among three patients who underwent kidney transplantation, all relapsed during the follow-up period (17, 29).

In our case, captopril was administered. Although there is no evidence suggesting that angiotensin-converting enzyme inhibitors (ACEi) are effective in the treatment of LPG, it is reasonable to use these drugs to retard the progression of renal disease in patients with persistent proteinuria (12). However, despite treatment, her lipid profile and serum creatinine were still abnormal after 2 years.

We have described a case of a typical LPG with *APOE* Kyoto mutation in a pediatric patient. Because LPG is difficult to distinguish from primary NS in pediatric patients, it is important to consider LPG as a differential diagnosis, especially in patients who have NS with atypical features (e.g., anemia), significant hypertension, family history of renal disease, or steroid resistance. Renal pathology and genetic testing may be crucial to diagnose the disease early and facilitate timely management.

## Author Contributions

YS, CY, and HW designed the study, and drafted and reviewed the manuscript. YS, CY, LL, and HW performed the literature search, made the tables, and reviewed the manuscript. YS and CY contributed equally to this work. All authors read and approved the final manuscript.

## Conflict of Interest

The authors declare that the research was conducted in the absence of any commercial or financial relationships that could be construed as a potential conflict of interest.

## Publisher's Note

All claims expressed in this article are solely those of the authors and do not necessarily represent those of their affiliated organizations, or those of the publisher, the editors and the reviewers. Any product that may be evaluated in this article, or claim that may be made by its manufacturer, is not guaranteed or endorsed by the publisher.

## Abbreviations

APOE: apolipoprotein E
LDL: low-density lipoprotein cholesterol
LPG: lipoprotein glomerulopathy
NS: nephrotic syndrome.
